# Influence of Pharmaceutical Direct-to-Consumer Advertisement on Medical Treatment of Inflammatory Bowel Disease—An Outpatient Survey-Based Study

**DOI:** 10.1093/crocol/otaa054

**Published:** 2020-11-07

**Authors:** Isaiah P Schuster, Hugo Mutz, Sapphire Perera, Royce Perera, Erin Taub, Ramona Rajapakse

**Affiliations:** 1 Division of Gastroenterology and Hepatology, Stony Brook University Hospital, Stony Brook, New York, USA; 2 Stony Brook University Hospital, Stony Brook, New York, USA; 3 Department of Medicine, Stony Brook University Hospital, Stony Brook, New York, USA; 4 Zucker School of Medicine at Hofstra, Northwell, Mather Gastroenterology, Port Jefferson, New York, USA

**Keywords:** inflammatory bowel disease, IBD, biologics, direct-to-consumer advertisement, surveys

## Abstract

**Background:**

The management of inflammatory bowel disease (IBD) has become much more complex as our understanding of the disease pathology has improved and as new novel therapeutic options come into play. A factor that has not been studied in the management of this disease is the role that Direct-To-Consumer Advertisement (DTCA) plays in patients’ decisions regarding their treatment options. Here we investigate the very role this mode of television advertisement has on influencing our patients.

**Methods:**

Following formal institutional review board approval, we devised a prospective, single institution, survey-based study in our university-based outpatient gastroenterology clinic. Surveys included major demographic features along with questions pertaining to patients’ interactions with various advertisements. Surveys were collected over a 3-month period.

**Results:**

Overall, 103 surveys were collected. The data were not normally distributed. Fifty-three patients were female, and 40 patients were male. Eighty-one percent of patients with IBD were not affected in any way by advertisements with regard to influencing their decision to start new therapies. A subgroup analysis revealed that various parameters including age, sex, and marital status played a role in how DTCA influences patients with regard to their IBD treatments.

**Discussion:**

This study demonstrates that overall patients are not significantly influenced by DTCAs; however, some cohorts are influenced in more ways than others. This study highlights the importance of understanding the role DTCA plays in influencing our patients with IBD and sets the foundation for further inquiry into this very interesting relationship.

## INTRODUCTION

The understanding and management of Inflammatory Bowel Disease (IBD) have significantly evolved over the last several decades. With the entry and use of antitumor necrosis factor agents such as Infliximab in the late 1990s, both clinicians and patients entered new frontiers in disease modification treatment.^1^ Since then, many more biologic agents and small molecules have been described and used in the management of complex cases of patients with IBD. Our understanding of treatment endpoints has evolved, with mucosal healing and prevention of complications being the major endpoint, in addition to improvement in symptoms and quality of life.^2^ Factors that influence the choice of therapy have expanded to include not only the extent and location of disease activity, presence of complications, and extraintestinal manifestations, but also patient-specific factors such as age, childbearing potential/desire, cancer history, insurance issues, and patient preference. It is clear that the treatment of IBD has to be individualized, with a push toward the use of biologics/immunomodulators early in those patients with aggressive disease.

The management of IBD has been further complicated as a result of patients having unprecedented exposure to a vast array of online and social media resources, spanning from forums written by licensed providers to patient-run support groups for generalized information and advice.^3^ Even when they are not seeking out specific groups, the average person watching television on any station is bombarded with advertisements for medications, including biologic agents. Now more than ever, direct-to-consumer advertisement (DTCA) is playing a tremendous role in patients’ lives. DTCA first emerged in the 1980s as a new and potentially potent form of advertising and is currently allowed in the United States, New Zealand, and Brazil and is steadily increasing revenue for pharmaceutical companies.^4–7^ Not surprisingly, patients now come to their outpatient clinic visits armed with pointers and specific medication requests as a result of advertisements they have seen or recommendations from Internet media.

There has been vigorous debate over whether there is an overall positive or predominantly negative effect of DTCA.^8^ Some authors argue that this form of media leads to misinformation and at times oversimplification, essentially clouding the healthcare landscape, and have called for a ban of DTCA.^8–10^ In contrast, some argue that DTCA has certain positive elements and helps motivate patients to bring their concerns forward to their providers.^8, 11^ Niederdeppe et al^12^ in their survey-based study in the early 2000s demonstrated that diagnosis of hyperlipidemia and statin use was overall higher in patients exposed to DTCA. It has also been demonstrated by many authors that overprescribing and DTCA have a close interrelationship, yet the reach of DTCA goes beyond medications and has been seen in the overutilization of cervical screening.^12–15^

With the development of new paradigms in IBD treatment, it is important to determine how DTCA affects patients. Identifying the degree of this influence can help clinicians better target patients’ concerns during what are often time-constrained visits. In this survey-based outpatient study, we aim to identify the effects of DTCA on patient’s decision making with regard to their IBD therapy after exposure to television-based advertisement. We also aimed to see how various demographic parameters (ie, marital status and sex) factor into the relationship between DTCA and IBD therapy. This study was not aimed to study specific agents or specific social media outlets. The insight gained in this study can help address preconceived notions about newer biologic therapies and their side effects, potentially allowing providers to start therapies earlier and/or tailor discussions with patients in the outpatient setting.

## METHODS

After receiving formal approval from our institutional review board at Renaissance School of Medicine at Stony Brook (IRB ID1244857-4), we performed a prospective survey-based study in our university-based outpatient Gastroenterology clinic on the north shore of Long Island, NY. Surveys were created using the Research Electronic Data Capture and were not based on any published surveys.^16, 17^

After informed consent was obtained, surveys were distributed to patients in the waiting area before their respective physician encounters. Basic demographic information was included first, followed by more specific questions regarding medical history, television habits, and prior medication history as well as questions specific to patients’ interactions with DTCA as given in Table 1. Research assistants were present to assist patients with the surveys and to collect them, thus ensuring compliance. The surveys were collected over a 3-month period and subsequently de-identified and is available in full in the Supplementary Material attached. Collected information included demographic data and duration of exposure to television, along with directed questions concerning exposure to DTCA. Information regarding prior biologic agents used by the patient and/or duration was not collected. All data were subsequently analyzed using SAS, version 9.4.^18^ Continuous variables were analyzed using Wilcoxon Rank Sum tests and categorical variables were analyzed using chi-square and Fisher exact tests. Likelihood ratios were also calculated with corresponding confidence intervals for questions regarding DTCA.

**TABLE 1.** Sample Survey Questions

Have you ever refused to take a prescribed drug for IBD based on its potential side effects?	[] YES [] NO
In the past year have you seen any television, magazine, online advertisements concerning for medications for IBD?	[] YES [] NO
Have you wanted to take a particular IBD medication after seeing an advertisement in an online, magazine, and/or television advertisement?	[] YES [] NO
Have you inquired about starting certain IBD medications, based on what you have seen in advertisements?	[] YES [] NO

## RESULTS

**FIGURE 1.**
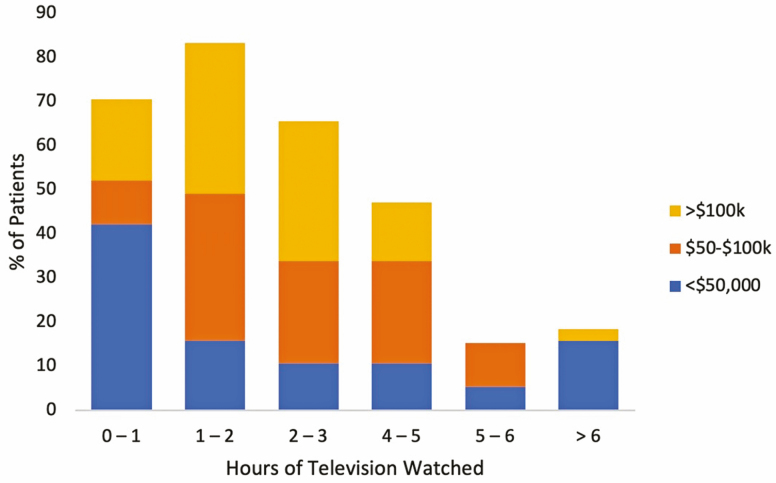
Association between income and hours of television watched.

A total of 103 surveys were collected over a 3-month period. The data were not normally distributed. Of all those surveyed, 53 patients were female and 40 patients were male with 10 individuals not filling out this segment of the survey. In addition, of the patients who did fill out the survey, 62% were employed, 6% were students, 7% were unemployed, 5% on disability, and 20% were retired. With regard to television exposure, the majority of patients only watched approximately 0–3 hours of television per day. Household income for most patients ranged from $75,000 to greater than $150,000. This was inversely related to the number of hours of television watched (*P* = 0.0196; Fig. 1).

**FIGURE 2.**
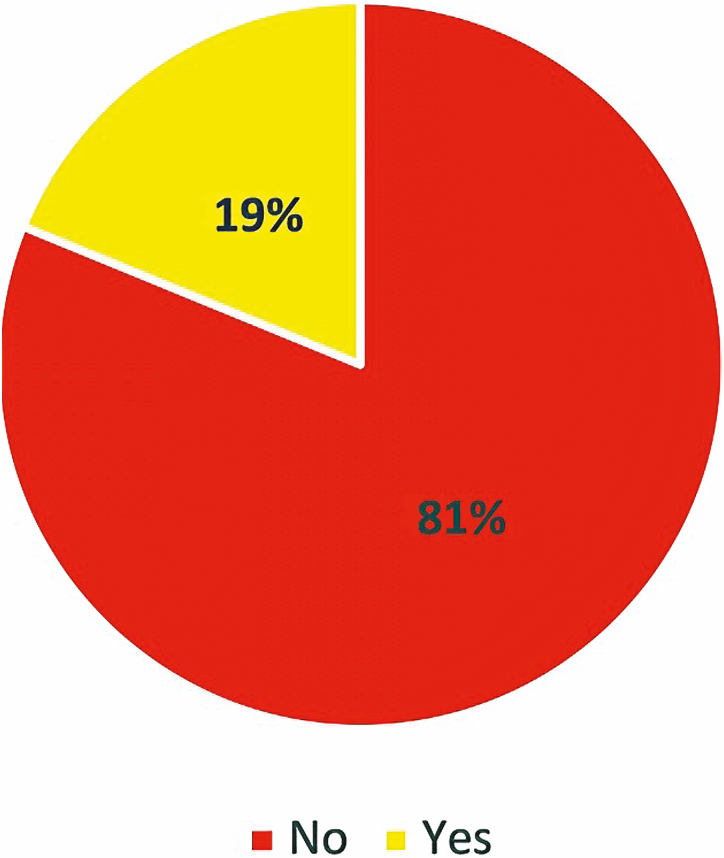
The overall effect of DTCA on willingness to initiate IBD medication based on the content of advertisement.

**FIGURE 3.**
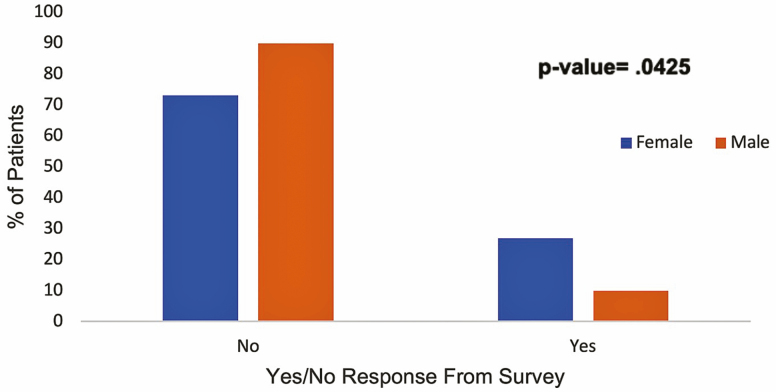
The subgroup analysis by sex—initiation of IBD medication after DTCA.

**FIGURE 4.**
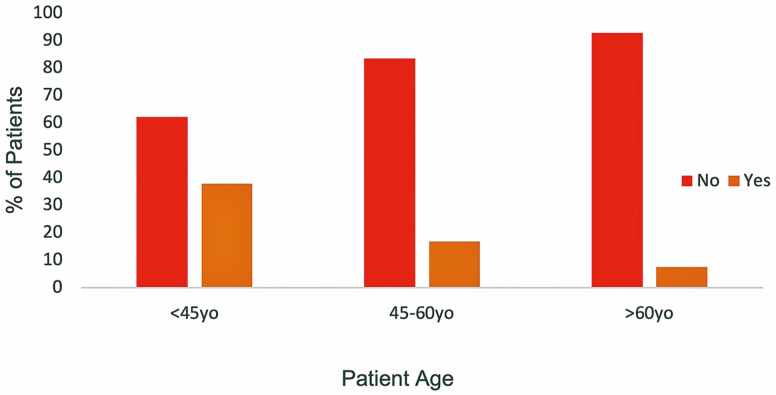
Relationship between age and starting IBD medication after DTCA.

Answers to DTCA-related questions showed that 81% of patients with IBD were not affected in any way by DTCA when it came to decisions regarding starting a new IBD medication compared to 19% of individuals who did demonstrate influence (Fig. 2). However, subgroup analysis revealed some interesting findings: There was a significant association between marital status and inquiry into starting a new IBD medication based on DTCA, with single patients having a higher percentage of responding positively than those who are married (*P* = 0.0330; relative risk [RR] = 2.7512, 95% confidence interval [CI] = 1.0582–7.1531). In addition, 26.95% of women compared to 10% of men were more likely to start a new IBD medication based on what they saw in a television advertisement and responded positively to that question (*P* = 0.0425; RR = 2.6923, 95% CI = 0.9993–7.5558; Fig. 3). Patients younger than 45 years were more likely to respond positively to starting a new medication following exposure to DTCA than older patients (*P* = 0.0102; RR = 2.9797, 95% CI = 1.3825–6.4225; Fig. 4).

## DISCUSSION

Although a number of studies have demonstrated the effect of DTCA on patients and providers in other subspecialties, to the best of our knowledge, this is the first study looking at the specific impact of DTCA in IBD. In an era where DTCA plays a large role in influencing patients’ knowledge and providers’ prescribing habits, it is critical to gain insight into the degree of impact to improve patient counseling and to deliver high-quality, individualized, patient-centered care.

In our small outpatient survey-based study, patients overall were not significantly influenced by DTCA, but certain cohorts were more affected than others. Younger patients were more likely to inquire about agents they had been exposed to in the media, and female patients were more likely to start new therapies based on their exposure to DTCA. The reason for the sex difference is unclear and difficult to determine but may be due to differences in overall media consumption. The fact that younger patients were more likely to make a change based on DTCA than older patients may be secondary to the duration of exposure to the advertising based on time spent watching television, potential feelings of immunity from side effects, and/or naivete and blind trust in media-related information.

This study highlights the importance of DTCA in patients with IBD and the influence that it may have beyond the physician–patient relationship. Although limited by its size and power, the preliminary information gathered in this study can be used as a stepping stone for larger studies to further identify the role DTCA plays in the decision tree of our patients with IBD. We need to consider the influence of media and advertising on our patients, especially when dealing with issues of noncompliance, due to fear of side effects or eagerness to start a new medication which may not be appropriate. Knowledge and understanding of these influences can help providers better address patients’ concerns and questions regarding their IBD treatment regimens. This is vital because early therapy with the right medication for the right patient can prevent complications and improve outcomes. This may become even more important in an era of patient-centered personalized care where tools that predict the onset of certain complications unique to each patient are being developed.^19^

It is clear that DTCA benefits the pharmaceutical industry. The majority of DTCA is focused on positive emotional appeals without significant information about the disease or a fair representation of alternatives.^11^ Ultimately, DTCA should serve our patients and help them make educated decisions about their care. Future directions should aim at understanding which forms of DTCA have the strongest impact on patients and how best to ensure that it is used with the best patient care as its primary goal. Of particular importance is the fact that DTCA is heavily embedded in social media and web-based app platforms which reach a younger, more vulnerable and influenceable audience. Moreover, it would be important to further delineate the specific elements of DTCA that have the most formidable impact on the patient consumer. Until we have larger studies, we urge clinicians to engage patients in a discussion regarding their biases about medications and where they arise from, in order to provide them with the best possible care.

## Supplementary Material

otaa054_suppl_Supplementary_Material

## Data Availability

All the figures in this manuscript were created using data obtained and analyzed from survey distribution. No new data was added in the revision of this manuscript.

## Notes

R.R. is the guarantor of the article.

Financial support: This study had no funding sources and did not use any sponsors for study design, collection, analysis, and interpretation and/or the writing of the manuscript. This work is entirely independent and investigator-driven.

Conflicts of interest and disclosures: None.
