# Supplementary material for: Influence of Pharmaceutical Direct-to-Consumer Advertisement on Medical Treatment of Inflammatory Bowel Disease—An Outpatient Survey-Based Study
Source: Crohns Colitis 360. 2020 Nov 7;2(4):otaa054. doi: 10.1093/crocol/otaa054 (PMC9802367; doi:10.1093/crocol/otaa054)
Supplement: otaa054_suppl_Supplementary_Material [file otaa054_suppl_supplementary_material.pdf]

Direct-To-Consumer Advertisement _ Patient Experience Survey

Study ID

____

Have you been diagnosed with inflammatory bowel disease, either Crohns Disease (CD) or Ulcerative Colitis (UC)?

- ◯ Yes
- ◯ No

Age (years)

____

Race

- ◯ American Indian/Alaska Native
- ◯ Asian
- ◯ Native Hawaiian or Other Pacific Islander
- ◯ Black or African American
- ◯ White
- ◯ More Than One Race
- ◯ Unknown / Not Reported

Gender

- ◯ Female
- ◯ Male

Marital status

- ◯ Single
- ◯ Married
- ◯ Divorced
- ◯ Other

ZIP Code

____

Employment Status

- ◯ Not employed
- ◯ Student
- ◯ Employed
- ◯ Disability
- ◯ Retired

Approximate household income (dollars per year)

- ◯ 0-25,000
- ◯ 25,000-50,000
- ◯ 50,000-75,000
- ◯ 75,000-100,000
- ◯ 100,000-150,000
- ◯ Greater than 150,000

Hours per day of television?

- ◯ 0-1
- ◯ 1-2
- ◯ 2-3
- ◯ 4-5
- ◯ 5-6
- ◯ Greater than 6

Have you or a family member ever experienced severe side effects from a prescribed drug for Inflammatory Bowel Disease (IBD)?

- ◯ Yes
- ◯ No

Have you ever refused to take a prescribed drug for IBD based on its potential side effects?	◯ Yes<br>◯ No
In the past year have you seen any television, magazine, online advertisements concerning medications for IBD?	◯ Yes<br>◯ No
If so, advertisements for which medications have you seen?	☐ Remicade (infliximab)<br>☐ Humira (adalimumab)<br>☐ Cimzia (certolizumab pegol)<br>☐ Tysabri (natalizumab)<br>☐ Entyvio (vedolizumab)<br>☐ Asacol/Lialda/Pentasa (mesalamine)<br>☐ Azulfidine (sulfasalazine)<br>☐ Dipentum (olsalazine)<br>☐ Entocort (budesonide)<br>☐ Other
Have you wanted to take a particular IBD medication after seeing an advertisement in an online, magazine, and/or television advertisement?	◯ Yes<br>◯ No
Have you had any major concerns about IBD medications, after seeing any media advertisement?	◯ Yes<br>◯ No
If so, which IBD medications were you concerned about?	______________________________
After having seen the television, magazine, and/or internet advertisement, are you now less likely to start or continue taking IBD medication?	◯ Yes<br>◯ No
Have you stopped taking prescribed IBD medication based on what you have seen in advertisements?	◯ Yes<br>◯ No
If so, which IBD medications?	______________________________
Has your doctor addressed concerns you may have had about IBD medications, particularly, after seeing something concerning via pharmaceutical advertisement?	◯ Yes<br>◯ No
Comments	______________________________
After seeing online, magazine, and/or television pharmaceutical advertisements concerning IBD medications, have you become more concerned about risks of developing any type of cancer, as a side effect?	◯ Yes<br>◯ No
Comments	______________________________
Have you inquired about starting certain IBD medications, based on what you have seen in advertisements?	◯ Yes<br>◯ No

Comments	______________________________
Do you think advertisements help patients with medication compliance?	◯ Yes<br>◯ No
Do you think online, magazine, and/or television commercial advertising help the physician in a financial way?	◯ Yes<br>◯ No
Comments	______________________________
